# Re-curating a 90-year ecological data collection from an Australian arid zone enclosure according to FAIR principles

**DOI:** 10.1038/s41597-026-07556-x

**Published:** 2026-06-08

**Authors:** A. Specht, M. Fittock, W. Karsdorp, A. Singh Ramesh, J. Sanchez Gonzalez, S. M. Guru

**Affiliations:** Terrestrial Ecosystem Research Network, the University of Queensland, Brisbane, Australia

**Keywords:** Environmental impact, Environmental sciences

## Abstract

This paper describes the re-curation of a 90-year legacy data collection from the TGB Osborn Vegetation Reserve in South Australia, first published by Dr Russ Sinclair in an open digital repository in 2014. Data collections such as this are rare, unrepeatable, and provide benchmarks against which the otherwise changing landscape can be measured. The work was necessitated by increasing sophistication in data repository systems, better understanding of the factors that make data reusable, demands of users, and the death of a key knowledge-holder. Updating a data resource that adhered to data standards of a decade ago, however, was not trivial. The data required cleaning and aligning with community standard vocabularies, with particular attention to the creation of comprehensive metadata. The lack of a knowledgeable custodian highlighted the need for an active relationship between the data creators and the repository. The updated dataset is now compliant with the FAIR Data Principles and provides an invaluable resource for the understanding and sustainable use of arid rangelands worldwide.

## Background & Summary

In this paper we describe the process of re-curating a long-term data collection available through the data repository of Australia’s Terrestrial Ecosystem Research Network (TERN, https://www.tern.org.au/). TERN, an infrastructure of the Australian Government through the National Collaborative Research Infrastructure Strategy (NCRIS: https://www.education.gov.au/ncris), provides both an observatory capacity for environmental scientists, and a repository for their data. The record that is the subject of this paper consists of a suite of biodiversity monitoring datasets from the Theodore George Bentley (TGB) Osborn Vegetation Reserve, a research enclosure established in 1926 in the rangelands of South Australia^1^. The site and its associated data collection have been managed since its inception by staff and students of Adelaide University. In the late 1990s, paper-based datasets from 90 years of various observations at the site began to be digitised by Dr Russell Sinclair and they were deposited in the TERN repository, the Australian Ecological Knowledge and Observation System (AEKOS), in 2014. These, together with the addition of new material, required updating to ensure compliance with the Findable, Accessible, Interoperable and Reusable (FAIR)^2^ Data Principles.

It is generally recommended that publicly funded research data be preserved and shared in a format that the research community can reuse. Long-term ecological datasets are vital for understanding environmental change as they contain observations from periods that cannot be reproduced^3–5^. Such datasets commonly were compiled in paper-based formats and perhaps digitised on local computers. Only in recent decades have datasets been stored in data repositories, but even then, often without reference to any of the original creators of the data. These records can be challenging to find and reuse, owing to the lack of good data management practices implemented throughout the data lifecycle^6,7^.

In recent years, the FAIR Principles for data and the TRUST (Transparency, Responsibility, User focus, Sustainability and Technology) Principles for digital repositories^8^, have provided clarity and guidance for researchers and data stewards to ensure research data (or other digital objects) are properly stored, discoverable and re-usable. Datasets created before such standards existed, however, will often require re-curation to enable effective discovery and reuse. For example, this may include updating metadata schemas to align with (i) standards such as Ecological Metadata Language (EML), Darwin Core (DwC) or Content Standard for Digital Geographic Metadata (CSDGM), and (ii) the application of community-accepted, standardised, domain-specific vocabularies. This relies on a partnership among data contributors, users, and the repository^9,10^.

The advancement of data management practices in the last decade and a culture of data reuse have increased the need for standardisation in a way that was unimagined in the early part of this century, let alone in the twentieth. The practicalities of ensuring that data holdings are maintained appropriately fall under the broad definition of repository custodianship. A data repository has to cope with constant updating and reviewing of data sets and needs the agility to adapt to changes in technology^6^. Gordon and Habermann^11^ explicitly mention the concept of maintenance (changes to the data tables or metadata, including update frequency) within their ‘discovery’ component, and Schirrwagen *et al*.^12^ include allowing the capacity for a repository to (i) make metadata edits, (ii) add objects, (iii) add or remove relationships between objects, and (iv) merge or split objects. Such curation activities may arise from improvements in technology in the repository infrastructure, and from realisation of inaccuracies in the data often flagged by the end-user. They may be considered necessary if they affect the discovery, the quality, and hence the trustworthiness of the data being held. In response to such revisions of data and metadata, and to the need for granular identification within time series data, it becomes increasingly important to record the revisions to or versions of the data when such changes occur^13^. The adoption of a ‘change log’ or ‘change history’ commonly used in software development provides a mechanism to record changes and to communicate and preserve the data provenance. This is not to be confused with new uploads of data as repeat measurements are made.

### The data source

**Fig. 1:**
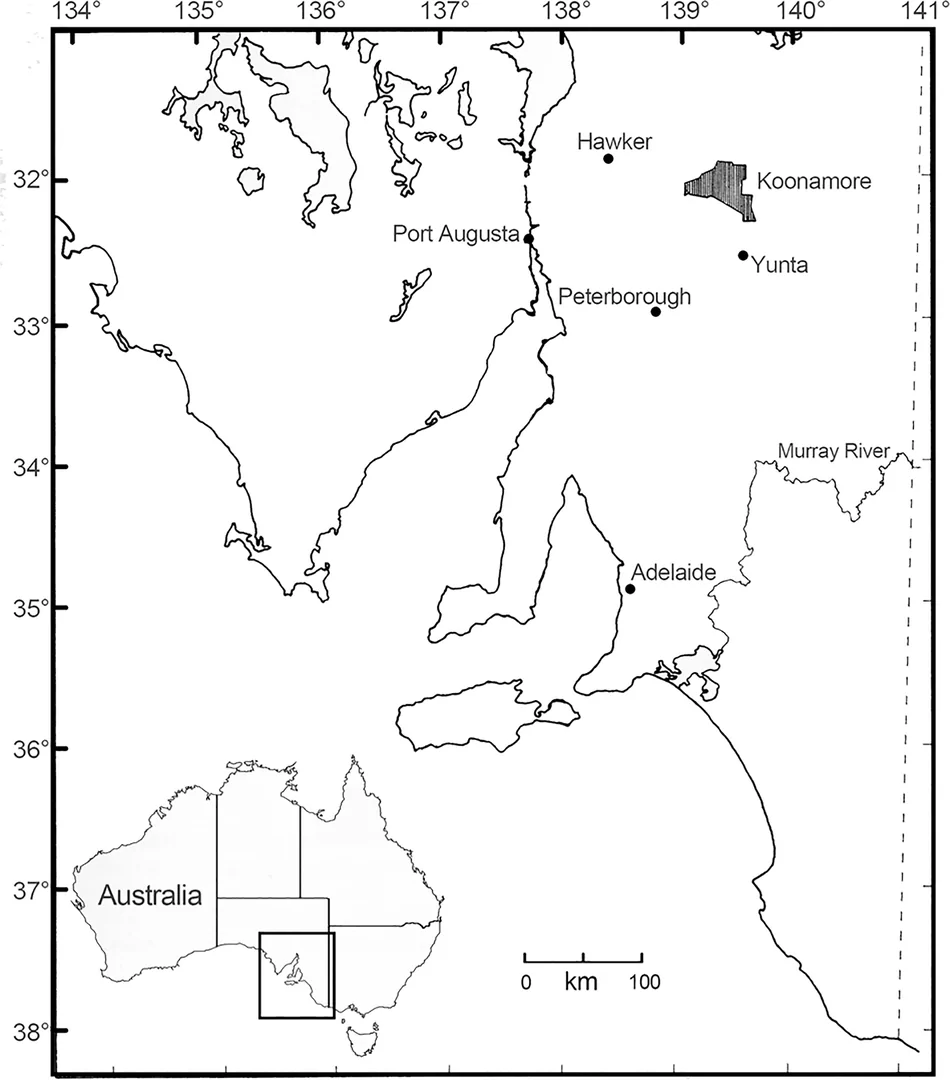
Location of Koonamore Station in South Australia, in which the TGB Osborn Vegetation Reserve is situated (map adapted from Fig. 1 of Hall *et al*.^17^).

In the mid-1920s Professor TGB Osborn, Professor of Botany in the University of Adelaide (now Adelaide University), identified a lack of knowledge of the ecology of arid zone vegetation in Australia and the effect on it of grazing^1^. As a result of Osborn’s strong advocacy, a parcel of land of around 390 ha was located within Koonamore Station in South Australia (Fig. 1) and fenced off to exclude rabbits and other stock. The area was originally referred to as the Arid Region Flora Reserve but was later named the TGB Osborn Vegetation Reserve after its founder. For simplicity, it is commonly referred to as the Koonamore Vegetation Reserve.

**Fig. 2:**
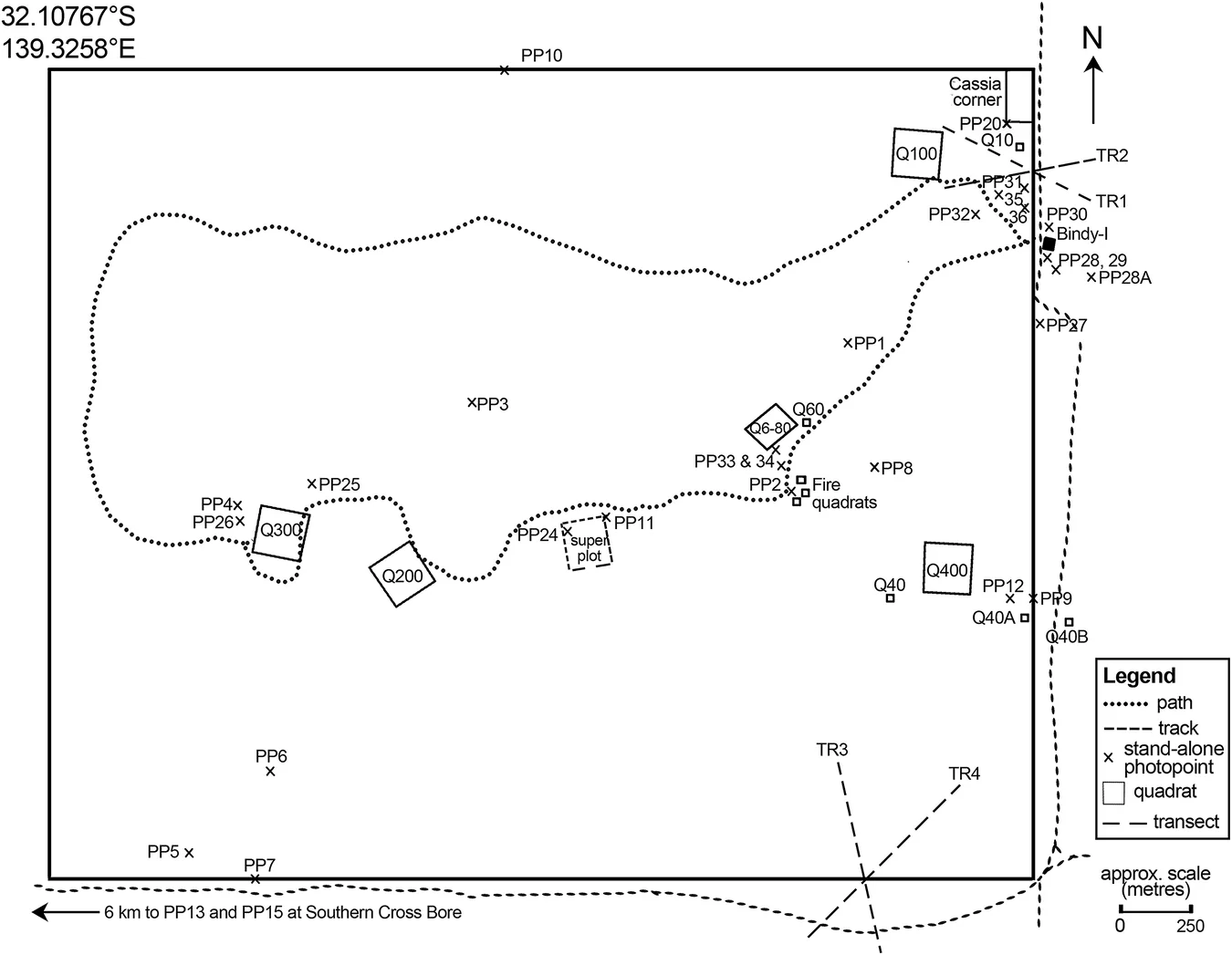
Sketch map of the type and distribution of measurements at the TGB Osborn Vegetation Reserve. Not all photopoint positions are shown, and the scale is approximate. Bindy-I is the field laboratory. The map is derived from those in Osborn *et al*.^44^ and Hall *et al*.^17^, an unpublished 1972 map, lists of georeferences for many of the locations, and Google maps. Reserve coordinates: NE corner: −32.10834° 139.35187°, SE corner: −32.12459° 139.35141°, SW corner: −32.12401° 139.32794°, NW corner: −32.10767° 139.32858°.

Systematic measurements were established in the now-protected vegetation. Osborn and his team set up a series of quadrats (square plots) and photopoints. A series of transects was later added (Fig. 2). All were permanently marked, and systematic (human) observations on plant species occurrence, physical measurements of the plants, and observations of kangaroo and rabbit activity were repeated over the century of existence of the reserve. These records were diligently kept in filing cabinets at Adelaide University throughout the lifetimes of several custodians, and many researchers and students. It is one of the longest-measured such sites in the world^14^. Since its inception, University students and staff have conducted repeat observations of the plants and animals at this site. After nearly eighty years of paper records, Dr Russ Sinclair of the University, with some assistance, gradually digitised the measurements and observations and published them in 2014 through AEKOS now superseded by the TERN Data Discovery Portal (TDDP: https://portal.tern.org.au/browse/theme).

There were eight ‘measurement types’ in the collection, vernacularly named: Vegetation quadrats, Photopoint data, Cassia corner, *Myoporum platycarpum* assessment, Kangaroo transects, Saltbush transects, Rabbit warren count, and Senna populations (Table 1). These types were retained throughout the updating procedure.

**Table 1:** Measurement types available through the AEKOS portal in 2014, arranged according to the date the measurements started.

Measurement type	Description
Vegetation quadrats (1926–2014)	Up to 76 repeat surveys of species within a suite of fixed plots (quadrats) within the Reserve: five large (100 m^2^), one 400 m^2^, eight 100 m^2^ plots, and four 1 m^2^ plots, as well as 13 ‘fire’ quadrats
Photopoint data (single observation in 1907, otherwise 1926–1988 with extension to 1994)	Approximately 108 temporal observations from 67 photopoints and six more observations from one location.
Cassia Corner (1940–2014)	Cassia (*Senna* spp.) abundance in a 1750 m^2^ rectangle on 26 occasions.
*Myoporum platycarpum* assessment (1955–2011)	Physical attributes of 25 individuals of *Myoporum platycarpum* on 21 occasions.
Kangaroo transect (1968–2014)	Kangaroo activity as evidenced by scat occurrence on 40 occasions both inside and outside the reserve.
Rabbit warren count (1977–2014)	Data from surveys of the reserve for rabbit activity for 56 occasions using the number of rabbit warrens (one warren indicated by 4 entrances) as evidence.
Saltbush transects (1989–2014)	*Atriplex* spp. occurrence from four paired transects on four occasions.
Senna populations (1997–2008)	The effect of three treatments (plus a control) to protect Senna seedlings from grazing in 10 instances.

By April 2025, based on work in the reserve, 55 journal articles had been published and nine postgraduate theses written^15^. One publication used the collated data that gave rise to the 2014 data collection^16^. Several drew on temporal data at periodic intervals (e.g.^14,17,18^), but most published on aspects of the animals and plants in the reserve, not the data collection *per se*. Enclosures like this, however, are vital for calibration and validation of remote sensing models^19^ but are not always cited.

On a number of occasions, the collection’s users have commented that the datasets were not understandable, accessible or reusable, certainly not meeting the requirements of the FAIR Data Principles. As a consequence, a review of the collection was initiated in late 2023 to update the data so researchers could more easily search, query and access individual observations. Although our focus was on making the existing data and metadata ‘more FAIR’, we also hoped that by doing so, we would provide a clear pathway for the data collectors to append their subsequent data to the collection.

## Summary

The re-curation of this legacy data collection has highlighted several matters. First, long-term data collections like this one are rare. They are historic and they cannot be replicated. They can be quite humble compared to today’s machine-collected data but they provide immense value to our understanding of environmental processes^3,18^. It was important to remember this as we progressed.

Second, although the initial upload was a package of datasets integrating a time-series of observations all in one place, considerable data housekeeping was required. The types of checking and revisions included data quality assurance, improving data organisation, adding related data previously unavailable due to technological limitations, updating vocabularies to present standards and updating metadata to enable better discovery. All necessitated evaluation of the data without reliance on the creator. A ‘change log’ was inserted into the metadata for each data package with the intent that this be updated as more data are added as per our intentions, or as adjustments are made in response to changes in technology and user expectations.

Thirdly, this is not a closed-loop confined to the repository and its data curators. In our experience, data collectors of long-term data can significantly enhance the scholarly record by (i) recognising that the data are as important as the field trip collecting them and the research publications resulting from them, (ii) allocating dedicated time into the project to ensure the data and records are digitised promptly and stored properly (interim data storage), (iii) ensuring there is a defined repository for the data for long-term preservation, and having an active relationship with that repository, and (iv) adhering to consistent frameworks and terminologies across time. The inevitable changes in data collectors and the shift in approaches that can occur in long-term sites always have demanded robust and standardised temporal records^20^. These demands can be offset by the establishment of rewards for participation in public-value data sets such as this one.

The work involved in this exercise was necessitated by a combination of increasing sophistication in data repository systems, better understanding of the factors that make data reusable, demands of users, and the loss of a key knowledge-holder and data enthusiast. There have been many developments in standardisation of metadata between 2014 and 2025, from attribute-level vocabularies through to expectations of the information provided for reusability. As has been clear for some time^6,21,22^, reviews and revisions such as those illustrated in this paper are likely to be necessary for all long-term data collections far into the future. Factoring such tasks into the workflow deserves consideration when deciding which data can be preserved over the long term.

## Methods

**Fig. 3:**
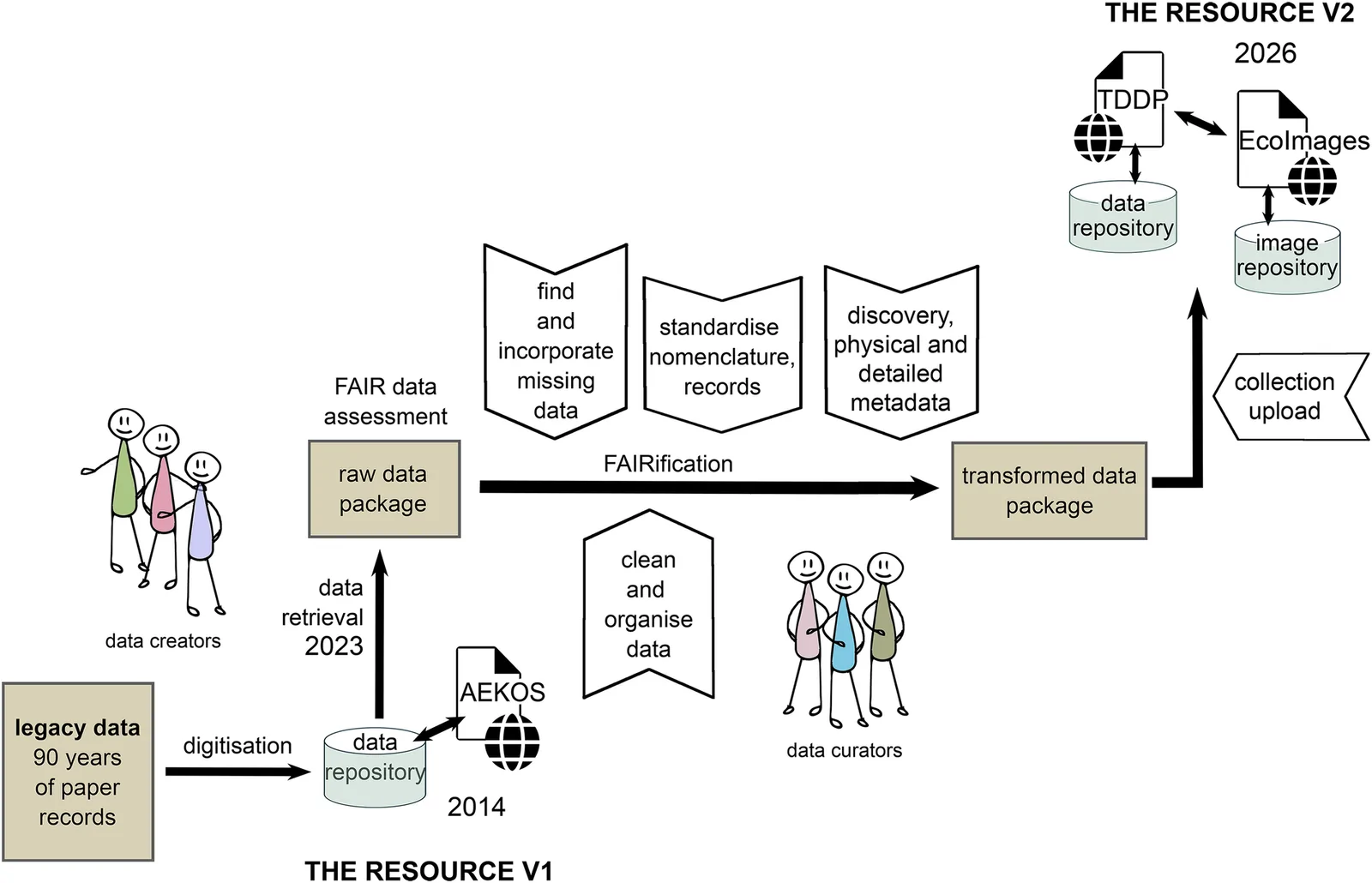
A flow diagram of the steps involved in creating the second version of the data collection from the TGB Osborn Vegetation Reserve, starting from the data in the AEKOS repository. The text follows this process. Key to abbreviations: FAIR: Findable, Accessible, Interoperable and Reproducible; AEKOS: Australian Ecological Knowledge and Observation System–an early repository of the Terrestrial Ecosystem Research Network (TERN); TDDP: the TERN Data Discovery Portal; EcoImages (https://ecoimages.tern.org.au/) the TERN Image access system.

The methodology described in this paper follows a stepwise approach starting with the retrieval of the data from the AEKOS repository in 2023 through to the delivery of a revised, FAIR-compliant data package in 2026 (Fig. 3). To determine the precise nature of any changes required, we needed to extract the available data from the repository, understand the datasets, obtain a cited manual, and liaise with the representatives of Adelaide University, Dr José Facelli and Mr David Ladd (hereinafter the ‘Adelaide team’). It should be noted that although the Adelaide team were involved in many of the field collection trips, Dr Sinclair was solely responsible for digitising the data from the original field sheets and for the data deposit in 2014. This was a considerable task, requiring great perseverance and sense of purpose. He was able to confer with several of the remaining data providers, such as Ray Specht^17^, but as is illustrated in this paper, he often had to make interpretations in cases where the creator was uncontactable. TGB Osborn, for example, died in 1973, and Constance Eardley, who for more than twenty years took students to the reserve, and supervised data collection, died in 1978. Dr Sinclair’s singular efforts have enabled these data to be secured in TERN, a certified CoreTrustSeal (https://www.coretrustseal.org/) repository for future reference by a broad audience.

### FAIR data assessment

A first review of the raw material showed that data uploaded in 2014 did not comply with the FAIR Data Principles in the following ways:

(i) Findability
  The data were discoverable through the AEKOS portal, but the geolocations given throughout were global, not specific to each measurement. The data were not directly associated with their metadata and it took several pathways to discover the metadata.
(ii) Accessibility
  No dataset and associated metadata were comprehensively located in one place, being variously distributed between the website and downloadable files. This required the user to move backward and forward from html-only web pages and the data files, or to transcribe the data manually.
(iii) Interoperability
  In 2014, there were several options for data download, from CSV to shape file, an H2 database and a Java SQL file, but none was comprehensive (as per (i) and (ii)) leading to heterogeneity and an inability to be integrated.
(iv) Reusability
  There was a repeated broad description of the history of the site across all datasets but no easy-to-read explanation of any particular dataset. Although collection methodologies were (mostly) described on the Internet, these relied on a cited but unobtainable manual.

In further detail, we found that all data required cleaning to verify the data quality, ensure terminological consistency, make text comments machine-discoverable, remove duplications, correct typological mistakes and update species names (Table 2). There were metadata updates to be made in all cases, as well as additions to the corpus such as the creation of data files from web-based texts, obtaining and publishing the photographs, links made between the photographs and the contemporaneous observations file (which was published in 2014, but without the photographs), separating key measurements from cells containing general comments to provide for machine readability, splitting datasets into different files in order to be consistent within each observation type, and detection and removal of duplicate data. Change logs also needed to be established in all cases.

**Table 2:** Commentary on the status of the datasets in 2014, and a broad statement of work needed to update to FAIR Data Principles.

Datasets	2014 status	Updating required
Vegetation quadrats (1926–2014)	Downloadable data were in two files: (a) vegetation populations, and (b) individual plant locations. The reason for and the overlap between the two was not clear. There were consistent anomalous subsamples within the population file. No locational information.	Due to differences in content, the vegetation population files need to be separated. The original files need cleaning. Data needs to be extracted from free text comments and made separately available. Geolocations need to be obtained and detailed metadata provided.
Photopoint data (single observation in 1907, otherwise 1926–1988 with extension to 1994)	The file of observations from photographs was intact, but the 67 geolocations were non-existent. No photographs were available for validation (or AI interpretation). The metadata did not explain the file structure making interpretation difficult.	A new observation file needs to be prepared to include geolocations. The file needs cleaning, removing duplication. Data needs to be extracted from free text comments and made separately available. The digital photographs need to be located, published, and linked to the observation file. Detailed metadata needs to be created.
Cassia Corner (1940–2014)	The area in which these data were collected was well defined. The data are a count, classification as one of two species, and whether adult or juvenile.	These data require little modification beyond general checking and the provision of detailed metadata.
*Myoporum platycarpum* assessment (1955–2011)	344 records of consistently identified individual plants. Geolocations not present.	Geolocations for each individual need to be obtained and added and detailed metadata provided.
Kangaroo transect (1968–2014)	The data were only on the web interface, and the downloadable file contained senna population data, not kangaroo data.	A new file for download (including geolocations) needs to be created, and detailed metadata provided.
Rabbit warren count (1977–2014)	These data were only obtainable as single-entry transcriptions from the web site.	A new file for download needs to be created and detailed metadata provided.
Saltbush transects (1989–2014)	These data were embedded in the vegetation populations file. Geolocations and direction of each transect was not clear from available material.	Due to differences in content, the data need to be separated and new files created. Geolocations and direction need to be clarified and added. Detailed metadata needs to be provided.
Senna populations (1997–2008)	These data were split between the web (requiring individual extraction) and a downloadable data set in the kangaroo package.	A new, dedicated, file for download (including geolocations) needs to be created and detailed metadata provided.

In all cases, we had to ensure that we complied with the requirements of the TERN Data Discovery Portal such as aligning with the TERN Controlled Vocabularies (https://linkeddata.tern.org.au/prez/tern-cv/v/).

### Find and incorporate missing data

The first author, who had historical familiarity with the site and the data, led this work.

#### Geolocations

One of the important steps was to obtain and verify the location details of the measurements (photopoints, quadrats, transects, individual plants). A few of these were held in an unpublished manual, but the majority were obtained from computer and GARMIN records held by Mr Ladd of the Adelaide team and subsequently confirmed and expanded upon by Dr Rebecca Greening. Where possible, geolocations were incorporated into the data files. This was able to be done for the photopoints, all but one Myoporum individuals, and the transects. The geolocations remain incomplete, however, especially for the smaller and older quadrats (some ceased being measured in 1931). In addition, although being quadrangular, the quadrats are not oriented in the same direction, and at least two corners would be required to register the orientation of the quadrat. We chose the north-east corner to be published in the CSV files. This was supplemented by a nearly-complete suite of quadrat and kangaroo transect georeferences which were published in a separate file and referenced in the metadata for the relevant data type^23^. This file was included in the collection documents.

#### Location maps

A map was created to explain the layout of the measurements in the reserve (Fig. 2). It is moreover valuable as it illustrates the range and type of activities at the site and the relative position of many of them. We felt that this map and Fig. 1 should both be included in the collection documents.

#### Photographs

A major search was initiated to find digital copies of the photographs. Prints and negatives were held in filing cabinets in the Biology Department of Adelaide University, but no scanned versions. It was felt that without the evidence of the images themselves, the dataset was inadequate. Having the images in digital format in a repository would also serve to preserve them. Fortunately, Dr Dean Graetz (retired) had digitised all the photographs, and he reported that they were on CDs at the University. Finding these CDs turned out to be a further issue. Eventually most of the images were found on the University’s server and copies were made. The CDs themselves were later discovered.

The existing photograph observation dataset (Table 2) was in the form of a spreadsheet of observations made from the images. These were listed according to photopointID alphanumeric codes that consisted of: (i) stand-alone photopoint number e.g. PP36 or PP4L, or (ii) quadrat or transect ID and position (e.g. ppQ10ANW or ppTR1PP5). The data contained within this spreadsheet were of species observed from the particular photopoint, and often, but not always, recorded physical attributes of the species (height, diameter, and general comments). These observations were made in the field or from the relevant photograph after returning to the laboratory; which of the methods used in each case was not stated. The direction in which the photograph was taken was not recorded anywhere and no-one could report how this was currently being done, but from observing the photographs it was clearly consistent. The geolocations for each photopoint were obtained and entered into the file. Other edits were made, such as checking the species names and correcting typographical errors. Nomenclature was made internally consistent and aligned with the other datasets. A large number of repeated rows in this dataset needed to be removed.

The sourced images included systematic quadrat, transect and photopoint photographs, but also included were photographs of intermittent verticals of ground quadrats, panoramas, non-replicated directional photos, experiments with different lenses, and photographs of people, signs, bogged cars, and infrastructure repairs. The advent of colour photography added to the variety of available images with the first colour photographs included in 1974 and 1977. With the exception of 1987, 1988 and 1998, when only colour photographs were taken, monochrome photographs were always paired with the colour ones until 2000. After 2000, only colour photographs were taken. As shown in Table 2, the images themselves extended later than the records available in the spreadsheet. The records, with one exception, finished in 1988. That exception was photopoint 1, which continued to be annotated until 1994. We made the decision to publish all images regardless of their appearance in the observation spreadsheet.

Each digital image was originally named as follows: filmnumber_photopointID_yyyymmdd.jpg. The film number referred to negatives stored in hard copy with the prints in filing cabinets. This format made it very difficult to sort according to the photopointID and date, and to separate images that were not directly related to the observational record. Our first step, therefore, was to remove the film number, for which we employed a Python script accompanied by human checking. The result was that many duplicate images were created with the same name, as without the film number there was frequently more than one image with the same name and date. To rectify this necessitated sequence numbers to be added to every image, but the process ultimately enabled us to organise the files according to type and date. We further corrected inconsistencies in image names at this point. After consultation across the team, the final file name was as follows: photopointID_yyyymmdd_seqN.jpg.

It was decided that a maximum of four images per photopointID and date be retained, ensuring a mixture of monochrome and colour images if both were present. At this point we removed images that were of very poor quality. Once this was done, we identified 5,889 images of relevance. These were listed in an Excel sheet facilitated again by a Python script and manually checked.

After the observations file was cleaned, four new columns were created to accommodate the names of the photographs relevant to a particular photopoint and date. These cells were populated using a Python script that recognised the root (photopointID) name and date in the measurement file and linked to the list of image names in the resource sheet. This process required several iterations, as minor typographical variances and inconsistencies were corrected in both files (observation measurements and image names). If there were fewer than four images for an event, ‘NA’ was entered into empty cells using an Excel function.

At this point, it became apparent that the date of the photograph did not always match the date of the measurement record, requiring decisions to be made about how to match the two. Sometimes the date on a photograph did not include day, only the month. Sometimes a photograph would be taken on a (slightly) different date from a measurement record; for example if the measurers forgot to take a photo and did so on the next trip. If the missing information or the difference in dates was not excessive, we manually aligned the image to the measurement record. The object of this was to permit the user to easily link the records in the data spreadsheet with the images available and vice versa.

### Clean and organise data

All the data files needed to be examined to identify superfluous material and determine necessary changes in formatting. The rationale in 2014 was to have uniform data fields for all vegetation datasets, whether relevant to a specific dataset or not. For example, of the 38 data fields in the 2014 vegetation populations file, thirty were not relevant^24^. Some fields had no values (a ‘0’). Some fields were duplicated with depositor ‘claims’ of scientific names and measurement dates kept in the files alongside repository-determined information. In the example, and indeed all the files, there was no difference in these two items rendering one entry superfluous. Repository-service tags, not relevant to the user, were also in the spreadsheets. Links to vital web-housed information meant the user had to be connected to the Internet to correctly interpret the files. Measurement units were in separate columns, further adding to the size of the spreadsheet. In 2014, the definition of each field (the data dictionary) was in the second row of the data file. This could be an advantage, in that there was no risk of disassociation of such information from the data, but the text in the cells was sometimes very lengthy and made the spreadsheet cumbersome. It is notable from the example that the 38 fields were reduced to eight (with one new field) in the final analysis, making the reuse of the data much simpler.

### Standardise nomenclature and records

The species names throughout were updated to current taxonomic nomenclature as stipulated by the Australian Plant Names Index (https://www.anbg.gov.au/apni/) and with reference to the Atlas of Living Australia (https://www.ala.org.au). Free-form comments in the cells of many of the files contained material such as X–Y coordinate references, species names, and life stage comments which we separated for machine-readability into their correct, separate columns.

The vocabularies in each data file of the collection were cross-matched and standardised to ensure harmonisation of field names throughout. The harmonised field names were then compared with the TERN Controlled Vocabularies and updated to standard names where possible. Parameters that were not listed in the TERN Controlled Vocabularies were matched with other global vocabularies such as the Environmental Thesaurus (ENVThes: https://github.com/LTER-Europe/EnvThes) and the Environmental Ontology (EnVO: https://github.com/EnvironmentOntology/envo). Where accepted, field names were adopted in the TERN controlled vocabularies using the Resource Description Framework (RDF) properties, such as closeMatch or relatedMatch. This ensured that the vocabulary was also standardised throughout the curation journey^25–27^.

### Discovery, physical and detailed metadata

Our aim was to facilitate reproducible research, for which we needed to ensure there was sufficient metadata^28^. Because we were working with a data collection rather than a singular dataset upload, we felt an introduction to the collection was required^29^. The created introduction covered the rationale for the establishment of the site, a summary of the datasets involved in the collection (very similar to Table 1 in this paper), and a species list for the site. A compilation of the species that occurred in each measurement type was made available separately^30^.

The general basis for understanding the measurements for each dataset (important for dataset evaluation by a user) was thought to exist in the cited, but not published, manual written by Dr Sinclair. When obtained from Dr Facelli of the Adelaide team, this manual was found to be a mixture of instructions for the field trips (food, safety, etc), and for often repeated measurement instructions, the latter gleaned from historic (1950s) as well as present practices. The manual was used alongside the AEKOS web commentary as the main resource for metadata creation. There were no physical or attribute-level summaries of the datasets available within the manual or otherwise, and these had to be added to the metadata for each dataset. A metadata statement is presented on the landing page for every downloadable object in the TDDP, and in addition to these statements, comprehensive metadata was provided as a downloadable file (after Gries *et al*.^10^) to ensure understanding of each data set and to reduce the chances of separation of vital contextual information from the data. Two of the most complex metadata files (for the vegetation quadrats^31^ and photopoints^32^) were eventually registered in the Zenodo^33^ general repository to enhance discovery.

### Upload into the TDDP

All materials have been uploaded to the TDDP and are available under a CC BY 4.0 license. The images are similarly available through TERN EcoImages. Integration of the collection into the TDDP and TERN EcoImages repositories required coordinated support from several data custodians at various stages; two staff members of the TERN data team—both of them authors of this paper—facilitated the image upload into TERN’s EcoImages Portal, and another staff member (also an author) checked the data and the uploading process. Additional data scientists and programmers supported the team.

For each data file, a corresponding data dictionary (CSV) and a supporting metadata file (PDF) were created, covering discovery level information, methodology, and attribute-level information. A change log was incorporated recording version changes^13^. The metadata documents were used to populate contextual metadata, including quality control processes compliant with the ISO 19115-3 metadata standard (https://www.iso.org/obp/ui/en/#iso:std:iso:19115:-3:ed-1:v1:en).

As mentioned, in addition to the metadata created for each data file, collection metadata was created, linking the various datasets and associated documents^29^. This was published to provide information on the temporal extent and frequency of collection, similar to that shown in Table 1, and was used both for the TDDP collection landing page as well as in downloadable form (PDF). Further supporting materials included a bibliography of the published literature derived from the reserve (to June 2025)^15^, a copy of the site maps (Figs. 1 and 2), the complete species list^30^, and a record of the available geolocations for the quadrats and kangaroo transect^23^.

The images and an associated spreadsheet listing image names were provided to the TERN EcoImages data custodians. The original images were archived in the QRIScloud (https://www.qriscloud.org.au/) to enable reprocessing for publication when required. The images were then uploaded along with image metadata enhanced by adding the species names from the spreadsheet. Using a test environment, the EcoImages framework was adjusted to accommodate the lack of provided directional information provided (North, South, East, West etc.), and the searchability of the portal was actioned to enable discovery by photopoint identification, date, or a combination thereof. Suites of images for a particular site could thus be sorted by date and downloaded, and the images across the site to be viewed date by date. The download includes the image metadata and citation details. Nearly 6,000 images are now discoverable through TERN EcoImages.

### FAIR data reassessment

Our original assessment of 2014 datasets highlighted several compliance gaps with the FAIR Data Principles. After responding to these gaps, we reassessed the datasets in terms of FAIR. The results are shown in Table 3.

**Table 3:** Advances against the FAIR Data Principles.

FAIR criteria	2014	2026
**Findability (discoverability)**	The data were easily discoverable through the (now deprecated) AEKOS portal. The geolocations given throughout, however, were only for the north-east corner of the reserve. There was a repeated broad description across all datasets without an easy-to-read description of the data set concerned. Although collection methodologies were (mostly) described on the web, these relied on an unpublished manual.	The updated data collection remains discoverable using the search term ‘TGB Osborn’ from the TDDP portal. Identification and discovery metadata about the collection and the various datasets associated with it are all on the landing page for each data set. Specific geolocations and maps are available. Documentation of the methods used to obtain the data are now incorporated in the metadata for each data set. Data are described in domain-specific metadata standards, and controlled vocabulary was used to describe all artefacts.
**Accessibility**	The data required for interpretation and analysis were distributed between a web platform and downloadable data. No data set was comprehensively obtainable in one place. The metadata were only found on the web site as html pages, and some data were also only found as html views. This required the user to move backward and forward from html-only web pages and the data files, or to type the data in manually.	The datasets and associated data dictionaries and metadata are all downloadable in CSV or PDF format from the same location
**Interoperability**	There were several options for data download, from CSV to shape file, and an H2 database, a Java SQL file, but none were comprehensive (as per the above) rendering them inoperable.	Field names, field values and vocabularies used in the files have been changed to align with the published TERN controlled vocabulary and international standards. Output format has been simplified to CSV or PDF format.
**Reusability**	The published data were not easily reusable due to confused data sets, the poor metadata or to the mode of delivery (i.e. only via html pages).	Reusability has been vastly improved due to improvements in all aspects of the metadata and the way the data are provided.

The exercise described proves that it is entirely possible to update old records to adhere with modern, FAIR, standards if there is sufficient effort and commitment by the repository and a dataset ‘custodian’. The importance of good, comprehensive, metadata cannot be over-stated. Much of the effort expended was in understanding the methodology and rationale that created the original, 2014, datasets. Naiveté in the original organisation and preparation of the data files with respect to reusability created considerable labour. We recommend that when submitting data for preservation and reuse, one must think very carefully about the re-users. What do they need to know? What detail, if missing, will make the data un-usable or not trustable? If data are not properly described and formatted, they may as well not exist.

## Data Records

**Fig. 4:**
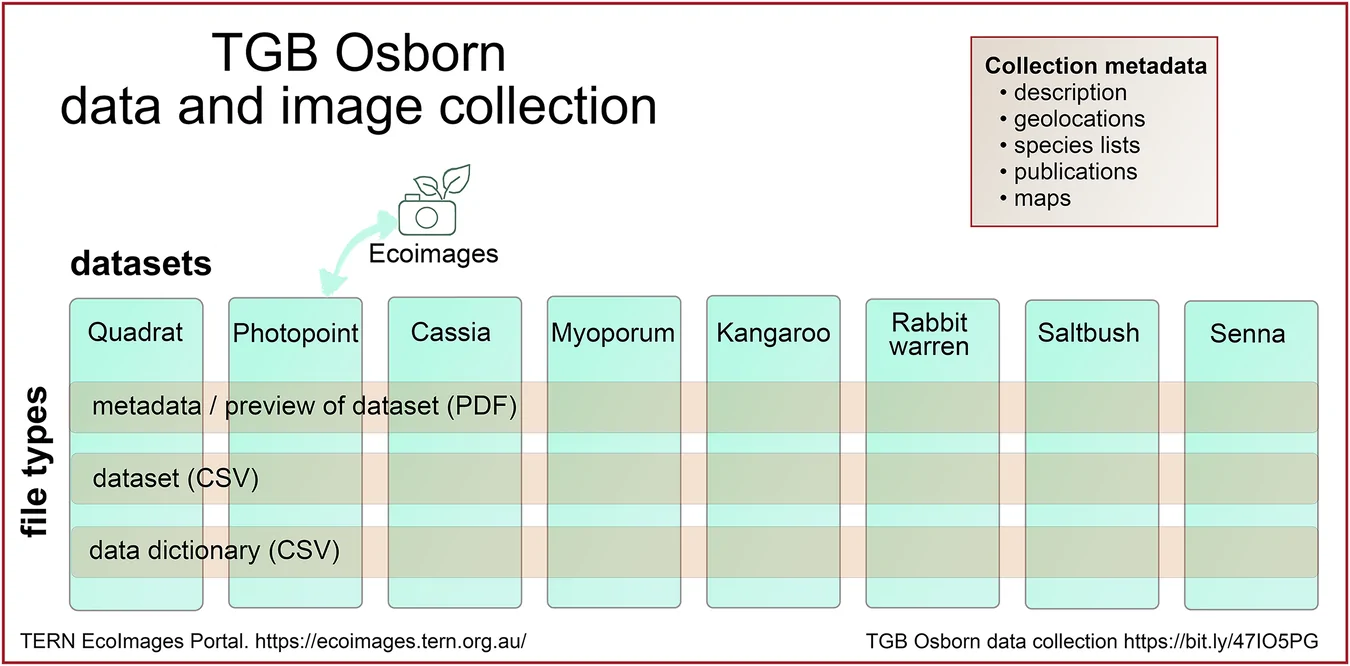
The dataset arrangement in the TDDP. The names of the data sets, if different from the shorthand in the diagram are as follows (L-R): ‘Vegetation quadrats’, ‘Cassia Corner’, ‘*Myoporum platycarpum* assessment’, ‘Kangaroo transect’, ‘Rabbit warren count’, ‘Saltbush transects’ and ‘Senna populations’. In a couple of instances (Vegetation quadrats and Saltbush transects) more than one data suite is present.

The TGB Osborn Vegetation Reserve data collection is available under a CC BY 4.0 licence through the TDDP. The categories of data records each contain two CSV files (a dataset and accompanying data dictionary) and one preview or metadata file explaining the nature of the datasets available in the category. A suite of collection materials (collection metadata) is included (Fig. 4).

Each category of data record is briefly described, with links, and ordered according to the date that each measurement started (Table 4).

**Table 4:** Datasets available through the TDDP for the TGB Osborn collection (https://bit.ly/47IO5PG) arranged according to date of inception as stated in Tables 1 and 2.

The TGB Osborn (Koonamore) data collection	
The collection is accessible through the TDDP portal https://portal.tern.org.au/browse/theme using the search term ‘TGB Osborn’ https://bit.ly/47IO5PG. The files are organised into folders according to subject.	
**Collection introduction**: https://doi.org/10.25901/jvty-sg24^35^	
Files (some files are also available on zenodo) include: • Metadata / preview (https://doi.org/10.5281/zenodo.18822119^29^), • Quadrat and transect geolocations (https://zenodo.org/doi/10.5281/zenodo.18930296^23^), • Publications to 2025 (https://doi.org/10.5281/zenodo.16946804^15^), • Species list (https://doi.org/10.5281/zenodo.18870470^30^), and • Location maps.	
**Vegetation quadrat data**	Up to 76 repeat surveys of species within a suite of fixed plots (quadrats): https://doi.org/10.25901/tqgh-vq42^36^
Files include the Metadata / preview (also available here: https://doi.org/10.5281/zenodo.18821933^31^), quadrat abundance data, quadrat individuals’ data and sub-quadrat data with associated data dictionaries.	
**Photopoint data**	Approximately 108 temporal observations from 67 photopoints: https://doi.org/10.25901/qajn-zy89^37^
Files include the Metadata / preview (also available here: https://doi.org/10.5281/zenodo.18821849^32^) and photopoint observations with associated data dictionaries. The relevant photopoint images are cited individually in the data files and the collection is found here: https://ecoimages.tern.org.au/search?site_id=kvr.	
**Cassia Corner**	Cassia (*Senna* spp.) abundance in a 1750 m^2^ rectangle on 26 occasions. https://doi.org/10.25901/4jr7-bb35^38^
Files include the Metadata / preview, and the Cassia Corner dataset and associated data dictionary.	
***Myoporum platycarpum*** assessment	Physical attributes of 25 individuals of *Myoporum platycarpum* on 21 occasions https://doi.org/10.25901/nxpr-eh69^39^
Files include the Metadata / preview, and the Myoporum individuals’ dataset and associated data dictionary.	
**Kangaroo transect**	Kangaroo activity as evidenced by scat occurrence on 40 occasions both inside and outside the reserve. https://doi.org/10.25901/sjr2-n626^40^
Files include the Metadata / preview, and the Kangaroo scat dataset and associated data dictionary.	
**Rabbit warren count**	Data from surveys of the reserve for rabbit activity on 56 occasions using the number of rabbit warrens (one warren indicated by 4 entrances) as evidence. https://doi.org/10.25901/nb1y-3e31^41^
Files include the Metadata / preview, and the rabbit warren count dataset and associated data dictionary.	
**Saltbush transects**	*Atriplex* spp. (*A. stipitata, A. vesicaria* and *A*. sp.) occurrence from four paired transects on four occasions. https://doi.org/10.25901/m3f1-hd14^42^
Files include the Metadata / preview, population, and individual datasets with associated data dictionaries.	
**Senna populations**	*Senna* sp. occurrence from four paired transects on four occasions. https://doi.org/10.25901/5wm1-px89^43^
Files include the Metadata / preview, the Senna dataset and associated data dictionary.	

## Technical Validation

To facilitate data validation, a password-protected online Google Drive folder was created where the data, once downloaded and/or created in spreadsheet form, was shared with the Adelaide team. Questions were posed through email about the content of the datasets, the metadata, and any anomalies. These messages were replicated in a sub-folder of the parent Google Drive. Status memoranda were provided for each dataset type throughout the process. This iterative approach supported part of the data validation, although it proved to be limited due to the lack of knowledge of the datasets by the Adelaide team. The lead author visited Adelaide three times for extended periods to discuss the challenges in person and to develop solutions. That such visits were necessary reinforced the need for adequate metadata to be supplied while fresh in the mind of the original creator.

Once the datasets had been initially cleaned and sorted, several of them were compared with published material (e.g. Lawley *et al*.^16^), to ensure no data anomalies were present. Some of the results of this process were presented at the TERN Science Symposium in 2025 and subsequently published^34^.

The TERN data team checked consistency across the data types, ensured the vocabularies and metadata were standardised, reviewed the files, and rectified any apparent lack of clarity.

## Data Availability

All the data are available under a CCBY 4.0 licence from the TDDP (https://portal.tern.org.au/browse/theme) using the search term TGB Osborn. A one-time login is required for every new user. This is required to monitor usage.

## Code availability

All code used to refine the datasets is available through the TERN Australia GitHub repository (https://github.com/ternaustralia/TGB-Osborn-VR).
